# Patient-Self Inflicted Lung Injury: A Practical Review

**DOI:** 10.3390/jcm10122738

**Published:** 2021-06-21

**Authors:** Guillaume Carteaux, Mélodie Parfait, Margot Combet, Anne-Fleur Haudebourg, Samuel Tuffet, Armand Mekontso Dessap

**Affiliations:** 1 Assistance Publique-Hôpitaux de Paris, CHU Henri Mondor, Service de Médecine Intensive Réanimation, F-94010 Créteil, France; melodie.parfait@gmail.com (M.P.); margotcombet@gmail.com (M.C.); annefleur.maignant@aphp.fr (A.-F.H.); samuel.tuffet@aphp.fr (S.T.); armand.dessap@aphp.fr (A.M.D.); 2 Groupe de Recherche Clinique CARMAS, Faculté de Santé, Université Paris Est-Créteil, F-94010 Créteil, France; 3 INSERM U955, Institut Mondor de Recherche Biomédicale, F-94010 Créteil, France

**Keywords:** patient-self inflicted lung injury, ventilator induced lung injury, acute respiratory failure, acute respiratory distress syndrome, artificial ventilation

## Abstract

Patients with severe lung injury usually have a high respiratory drive, resulting in intense inspiratory effort that may even worsen lung damage by several mechanisms gathered under the name “patient-self inflicted lung injury” (P-SILI). Even though no clinical study has yet demonstrated that a ventilatory strategy to limit the risk of P-SILI can improve the outcome, the concept of P-SILI relies on sound physiological reasoning, an accumulation of clinical observations and some consistent experimental data. In this review, we detail the main pathophysiological mechanisms by which the patient’s respiratory effort could become deleterious: excessive transpulmonary pressure resulting in over-distension; inhomogeneous distribution of transpulmonary pressure variations across the lung leading to cyclic opening/closing of nondependent regions and pendelluft phenomenon; increase in the transvascular pressure favoring the aggravation of pulmonary edema. We also describe potentially harmful patient-ventilator interactions. Finally, we discuss in a practical way how to detect in the clinical setting situations at risk for P-SILI and to what extent this recognition can help personalize the treatment strategy.

## 1. Introduction

After several decades of experimental and clinical research, clinicians have become familiar with the concept of ventilator induced lung injury (VILI) [1], i.e., the possibility that lung injury is induced or worsened by artificial ventilation. The understanding, even partial, of the main pathophysiological mechanisms leading to VILI have been translated in the clinical setting by the implementation of protective ventilation, which has been successful to decrease the mortality in the acute respiratory distress syndrome (ARDS) [2,3,4]. In recent years, the new concept of patient-self inflicted lung injury (P-SILI) has emerged, i.e., the possibility that lung injury is induced or worsened by the patient’s own inspiratory effort [5]. This concept seems interesting because it is based on sound physiological reasoning [6,7,8,9,10,11], an accumulation of clinical observations [12,13,14] and some consistent experimental data [15,16]. However, to date, no clinical study has validated its relevance. Thus, the way in which it should be taken into consideration in therapeutic choices remains a matter of debate [17,18]. Indeed, the therapeutic implication of this concept is potentially major, because if the patient is endangered by his own inspiratory effort, then controlled mechanical ventilation can become a protective treatment.

The purpose of this review is to present the main pathophysiological mechanisms by which the patient’s respiratory effort could become deleterious, and to discuss the potential clinical implications.

## 2. Inspiratory Effort and Mechanical Forces Promoting Lung Injury

One of the main mechanisms of VILI is excessive strain [1], which is defined as the ratio between the tidal volume and the initial lung volume, i.e., the end-expiratory lung volume [19]. Excessive strain corresponds to over-distension. ARDS is typically at high risk of excessive strain due to the dramatic reduction in end-expiratory lung volume [20]. Tidal volume reduction is therefore the emblematic intervention for protective ventilation, which has been shown to reduce mortality during ARDS [2]. The force that produces the strain (i.e., the deformation of the lung), is the stress. Physiologically, the stress corresponds to the transpulmonary pressure (P_L_), which is the transmural pressure of the lung, defined as the difference between alveolar and pleural pressure [21,22]. Tidal volume can therefore be delivered either by increasing the airway pressure—as during controlled positive pressure ventilation—or by decreasing the pleural pressure—as during spontaneous breathing or negative pressure ventilation (iron lung). 

In a seminal experiment, Dreyfuss et al. ventilated rats in three different modalities: with an artificial ventilator delivering high airway pressure resulting in high tidal volumes, with an iron lung generating large negative intra-alveolar pressure (mimicking spontaneous ventilation) resulting in similarly high tidal volumes, and finally with an artificial ventilator delivering high airway pressure but low tidal volume due to prior thoracoabdominal strapping of the animals [15]. The authors reported that pulmonary oedema developed only in rats ventilated with high tidal volumes, regardless of how the strain was generated, using a positive or a negative pressure.

In another experiment published 30 years ago, Mascheroni et al. repeatedly injected sodium salicylate into the cisterna magna of healthy sheep to generate a central acidosis, causing major sustained hyperventilation [16]. Hypoxemia developed within 12 h, compliance deteriorated within 24 h, and, at necropsy, the lungs showed macroscopic atelectatic areas and the lung/body weight ratio was increased. Such lesions were not observed in a control arm in which sodium salicylate was injected in the same manner but the sheep were sedated, paralyzed, and ventilated with normal tidal volumes.

Thus, a strong inspiratory effort can lead to excessive strain, which carries the same risk as high tidal volume delivered by positive insufflation from an artificial ventilator. This is the most straightforward pathophysiological mechanism of P-SILI.

## 3. Physiological Effects of Inspiratory Effort at a Given Global Transpulmonary Pressure

The same P_L_ generated by fully controlled mechanical ventilation, assisted mechanical ventilation or spontaneous breathing leads to the same tidal volume [6]. However, the participation of the inspiratory effort in P_L_ has its own physiological effects, especially in case of strong effort applied to an injured lung.

### 3.1. Stress and Strain Inhomogeneity

**Figure 1:**
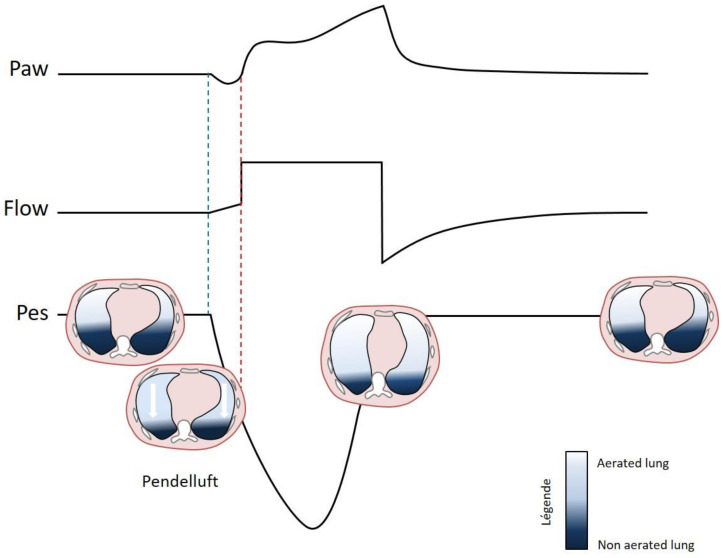
Schematic illustration of the pendelluft phenomenon. Pendelluft corresponds to an intrapulmonary shift of gas from the non-dependent anterior regions to the dependent posterior regions at the very onset of the inspiratory effort (just after the onset of the deflection on the esophageal pressure curve (Pes), blue dashed line), even before the beginning of the ventilator’s insufflation (red dashed line). Thus, with no change in overall volume, there is an abrupt loss of aeration in the anterior regions, and a concomitant increase in aeration in the posterior ones. Definition of abbreviations: Paw: airway pressure; Flow: airway flow; Pes: esophageal pressure.

During volume assist-control ventilation, the tidal volume, and thus the overall transpulmonary pressure variation, remains constant whether there is an inspiratory effort or not. In a model of ARDS in rabbits under volume assist-control ventilation, Yoshida et al. reported that high inspiratory effort was accompanied by an inhomogeneous distribution of transpulmonary pressure variations across the lung [7]. In fact, during inspiratory effort, regional transpulmonary pressure changes were much greater in the dependent (posterior) regions than in the nondependent (anterior) ones. These large regional forces applied to the posterior regions were accompanied by a pendelluft phenomenon, which corresponds to an intrapulmonary shift of gas from nondependent to dependent lung regions at the very onset of inspiratory effort, even before the start of ventilator insufflation (Figure 1) [11]. The consequence was to significantly increase the cyclic inflation of the dependent regions which were collapsed during expiration, thus reproducing another major mechanism of VILI, the so-called atelectrauma [23]. Pendelluft also exposed dependent regions to regional volutrauma irrespective of the size of the insufflated tidal volume. The regionalization of transpulmonary pressure variations and its effects on regional ventilation (pendelluft, cyclic opening/closing) disappeared after muscle paralysis [7,11]. Chest electrical impedance tomography measurements in ARDS patients under assist-control ventilation found the same effects of spontaneous ventilation on regional ventilation [7,11].

### 3.2. Alveolar Pressure

**Figure 2:**
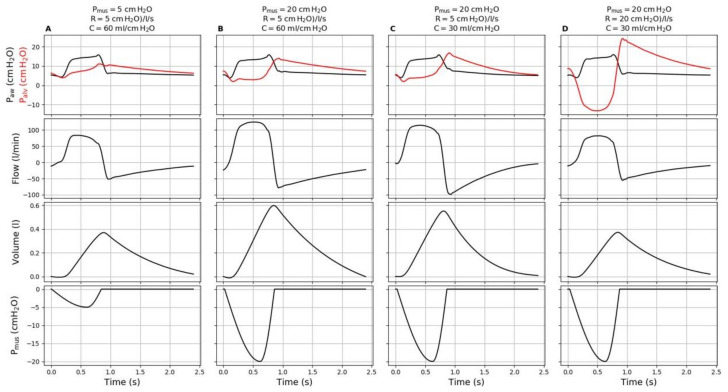
Combined effects of inspiratory effort and respiratory mechanics on tidal volume and alveolar pressure during pressure support ventilation. A ventilator set in pressure support ventilation with a PEEP level of 5 cm H_2_O and a pressure support level of 10 cm H_2_O was connected to an active lung simulator that simulated various respiratory system compliance (C), resistance (R) and muscle pressure (Pmus). Note that Pmus of 5 and 20 cm H_2_O correspond to esophageal pressure swings (∆Pes) around 3 and 18 cm H_2_O. When simulating normal respiratory mechanics and low inspiratory effort (**A**), the tidal volume was within usual clinical range and alveolar pressure (Palv, red line) remained above the PEEP. Marked increases in inspiratory effort (**B**) resulted in important increase in tidal volume and decrease in alveolar pressure below the PEEP. Thus, the decrease in respiratory system compliance with the same strong simulated inspiratory effort (**C**) yielded a decrease in tidal volume without changing that much the alveolar pressure. Finally increasing the resistance (**D**) led to an important decrease in tidal volume and a dramatic drop in alveolar pressure far below 0 cm H_2_O. Note that of these four conditions, the one that represented the lowest risk of P-SILI (A: normal respiratory mechanics, low inspiratory effort, alveolar pressure above the PEEP) and the one that represented the highest risk of P-SILI (D: impaired respiratory mechanics, intense inspiratory effort, fall in alveolar pressure far below zero) led to similar tidal volumes and comparable flow and airway pressure waveforms. Thus, the detection of a situation at high risk of P-SILI can be particularly difficult during pressure support ventilation and may be facilitated by esophageal pressure monitoring and careful clinical assessment. Definition of abbreviations: Paw: airway pressure; Flow: airway flow.

During controlled ventilation, alveolar pressure is constantly greater than or equal to the positive end expiratory pressure (PEEP). During assisted ventilation, significant inspiratory effort can be accompanied by a fall of the alveolar pressure below the PEEP, particularly for low levels of assistance [6]. This drop in alveolar pressure can be greatly amplified when airway resistance increases (Figure 2) [6,24]. Such a dramatic decrease—potentially far below zero—in alveolar pressure in case of high inspiratory effort and increased resistance is likely to increase the transvascular hydrostatic pressure and thus favor the aggravation of pulmonary edema [9,24,25,26,27], especially in case of prior alteration of the alveolo-capillary membrane.

All in all, for the same variation in transpulmonary pressure, the participation of inspiratory effort may be accompanied by potentially deleterious mechanisms. It is, however, important to keep in mind that most of such mechanisms have been described when a strong inspiratory effort is imposed to a previously severely injured lung. As an illustration, in an experimental model of ARDS, Yoshida et al. reported worsening of lung damage with high inspiratory effort and severe prior lung injury [28]. In contrast, in animals with mild lung injury, inspiratory effort was accompanied by an improvement in lung injury.

## 4. The Vicious Circle of P-SILI

Patients with injured lungs usually have a high respiratory drive because of gas exchange and respiratory mechanics impairment [29,30]. Thus, providing that the neuromuscular transmission is preserved, high respiratory drive results in strong inspiratory efforts, which may be accompanied by the physiological effects described above (risk of over-distension, pendelluft, atelectrauma, and increase in vascular transmural pressure) and are, therefore, likely to aggravate the pre-existing lesions. This additional worsening of the gas exchange and respiratory mechanics will lead to an even higher respiratory drive, which in turn will expose the lungs to the risks of even stronger inspiratory efforts. Thus, the concept of P-SILI includes a dynamic dimension that acts as a vicious circle [5] that must be broken. Invasive controlled mechanical ventilation could then emerge as a protective and preventive treatment of P-SILI.

## 5. Patient-Ventilator Interaction

From a pragmatic standpoint, P-SILI pathophysiology should also address situations in which inspiratory effort is not necessarily dangerous per se but becomes so when associated with mechanical ventilation.

During pressure support ventilation, the synchronous addition of the pressure support level to an already important negative swing in pleural pressure in patients with a high respiratory drive can further increase the transpulmonary pressure and its subsequent risk of over-distension [31]. In case of over-assistance, the occurrence of ineffective triggering have not been proven to increase the risk of P-SILI but may expose the diaphragm to potentially injurious eccentric contractions. Conversely, under-assistance carries the risk of dangerous increase in inspiratory effort that may increase transvascular pressure and promote pendelluft and regional over-distension [32].

During volume assist–control ventilation, the patient’s neural inspiratory time is usually greater than the ventilator’s insufflation time. Thus, when the inspiratory effort duration is prolonged beyond the insufflation’s end, it can immediately trigger a second insufflation, leading to a double triggering (also called breath stacking) [33,34]. During such asynchrony, the patient receives twice the tidal volume.

During controlled ventilation, the phenomenon of reverse triggering has been described, which corresponds to an inspiratory effort occurring after a ventilator-initiated breath, as if the diaphragm contraction was triggered by the ventilator’s insufflation [35]. Reverse triggering can be associated with increase in transpulmonary pressure during pressure-control ventilation [36] and can frequently result in breath stacking, which may cause large tidal volumes [35,36]. Additionally, some clinical observation and experimental data suggest that inspiratory effort during reverse triggering may also lead to potentially deleterious inflation of dependent regions as previously described, even in the absence of breath stacking [8].

## 6. Usefulness of the Concept of P-SILI in Clinical Practice

Transposed to the clinical setting, the concept of P-SILI can help guide patient assessment to discriminate potentially hazardous situations for the lung, such as the combination of excessive inspiratory effort and severe lung injury. However, this evaluation must also balance the risk of diaphragm atrophy and weakness due to excessive muscle unloading [37,38,39,40]. As both excessive and insufficient respiratory effort may result in deleterious anatomical and functional modifications of the diaphragm [41], lung- and diaphragm-protective ventilation can often be reconciled by promoting a “normal” inspiratory effort, consistent with resting breath [42,43]. Additionally, it is important to bear in mind the other beneficial effects of preserving inspiratory effort (i.e., protection against expiratory airway closure and atelectasis [44], reduction in the need for sedation and easier mobilization), and that the weaning process requires early spontaneous ventilation [45]. However, if there is a conflict between diaphragm and lung protection, it is widely recognized that lung protection must be prioritized.

To achieve lung- and diaphragm-protective ventilation, it is therefore, necessary to assess inspiratory effort and characterize patient–ventilator interactions. Whenever inappropriate inspiratory effort is detected, physiological and clinical reasoning at the bedside should be conducted taking into account the severity of the underlying lung injury, and systematically looking for situations where excessive respiratory drive is independent of the respiratory load, e.g., metabolic acidosis and uncontrolled pain [29,30].

### 6.1. Measurement of Respiratory Effort

Reliable assessment of the respiratory effort can be achieved by recording the esophageal pressure (Pes) signal as an estimation of the pleural pressure using an esophageal catheter [21,22]. Pes allows direct quantification of respiratory effort indices, such as muscle pressure (Pmus, which corresponds to the pressure generated by the inspiratory muscles and is computed as the difference between the static recoil pressure of the chest wall and the swing in Pes), esophageal pressure-time product (PTPes, which represents the integral of the Pmus curve over time), work of breathing (WOB, computed as the integral of the product of Pmus and the generated volume), but also to calculate the P_L_ [21,22]. A normal or acceptable level of respiratory effort under mechanical ventilation is usually considered as a Pmus between 5 and 10 cm H_2_O [22,46], a PTPes between 50 and 150 cm H_2_O·s·min^−1^ [22,46,47], a WOB between 2.4 and 7.5 J·min^−1^ or 0.2 and 0.9 J·L^−1^ [21]. In the clinical setting however, these indices are usually difficult or impossible to monitor and are instead computed offline for research purpose. The inspiratory swing in esophageal pressure (∆Pes) is much easier to measure at the bedside, and can be directly monitored on the screen of some ventilators. With the chest wall compliances and tidal volumes usually observed, the ∆Pes absolute value is approximately 2 ± 1 cm H_2_O lower than the Pmus absolute value. Thus, Pes recording allows detecting inappropriate inspiratory efforts. A ∆Pes absolute value lower than 2 to 3 cm H_2_O is usually considered as a sign of over-assistance. Conversely, a ∆Pes absolute value higher than 8 to 12 cm H_2_O is considered as a marker of under-assistance [42]. Esophageal pressure monitoring also offers the possibility to monitor the dynamic transpulmonary pressure and transpulmonary driving pressure by performing inspiratory and expiratory hold during assisted ventilation [42]. However, the use of Pes in the clinical setting remains limited as it is often seen as a research tool dedicated to expert centers.

The diaphragm electrical activity (EAdi) can be obtained using a specific eso-gastric tube and a dedicated ventilator. EAdi is a surrogate of respiratory drive and is tightly correlated with the respiratory effort indices described above [47,48,49,50]. Because of a wide interindividual variability in the proportionality between EAdi and respiratory effort indices, no specific threshold values for over- or under-assistance can be defined. However, the proportionality coefficient between EAdi and Pmus can be calculated from airway pressure drop during end-expiratory occlusions, making feasible to estimate the Pmus value from the EAdi value [51,52]. Monitoring of EAdi is also useful for detecting patient-ventilator asynchronies. It is, however, rarely accessible in clinical practice. The tools described below are much more widely available at the bedside.

Airway occlusion pressure (P_0.1_), which corresponds to the negative variation in airway pressure generated by an airway occlusion during the first 100 milliseconds of the inspiratory effort, is used as an index of respiratory drive [29,30,53]. Its value in healthy subjects varies between 0.5 and 1.5 cm H_2_O. During assisted mechanical ventilation, a P_0.1_ > 3.5 cm H_2_O allows the detection of excessive inspiratory effort with a sensitivity of 80% to 92% and a specificity of 77% to 89% [52,53]. Interestingly, P_0.1_ measurement is now available on the latest generation of mechanical ventilators, albeit with varying degrees of accuracy [53,54], and can therefore, be used to assess the respiratory drive and detect excessive inspiratory effort at the bedside.

The negative swing in airway pressure generated by respiratory muscle effort during a single-breath expiratory occlusion (Pocc) can be used to detect excessive inspiratory effort or excessive dynamic transpulmonary pressure [55,56], with a threshold value of −15 to −20 cm H_2_O [42].

Diaphragm ultrasound can be used to assess patient-ventilator interactions and quantify the thickening fraction (TFdi), which correlates with inspiratory effort [57]. A TFdi below 15% can be considered as a sign of over-assistance and a TFdi above 30% to 40% as a sign of under-assistance [42].

Lastly, one specific partial ventilatory mode, proportional assist ventilation with load-adjustable gain factors (PAV+), displays continuous monitoring of the respiratory mechanics [58,59] and offers the possibility to calculate Pmus without the need for Pes [46,60].

### 6.2. Clinical Situations at Risk of P-SILI

When Pes monitoring and measurement of respiratory effort indices are not routinely performed, daily careful analysis of clinical data and ventilator’s waveforms should be performed in order to detect situations at risk of P-SILI. We will now discuss a non-exhaustive list of such situations as well as interventions that may limit the risk.

#### 6.2.1. Detecting a Risk of P-SILI in Volume Assist-Control Ventilation

**Figure 3:**
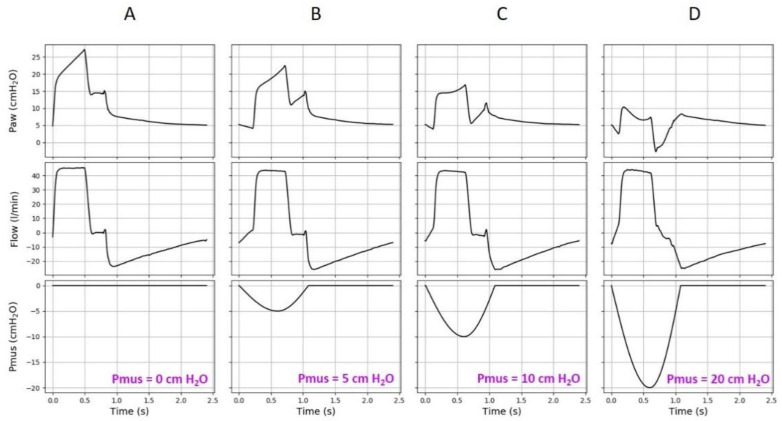
Effect of inspiratory effort on the airway pressure waveform during volume assist-control ventilation. A ventilator set in volume assist-control ventilation was connected to an active lung simulator. Passive conditions were simulated (**A**), followed by increasing inspiratory effort with a simulated muscle pressure of 5 (**B**), 10 (**C**), and 20 (**D**) cm H_2_O. As inspiratory effort increased, the airway pressure decreased and the waveform became “deeper”. This phenomenon makes it possible to detect intense inspiratory efforts by analyzing the ventilator’s waveforms at the bedside. Note that a Pmus of 5, 10 and 20 cm H_2_O correspond to an esophageal pressure swings (∆Pes) around 3, 8 and 18 cm H_2_O. Definition of abbreviations: Paw: airway pressure; Flow: airway flow; Pmus: muscle pressure of the inspiratory muscles.

During assist-control ventilation, the detection of a strong inspiratory effort can be done by simple inspection of the airway pressure waveform. Volume assist–control ventilation is characterized by constant insufflation flow rate and tidal volume delivery. Thus, by definition, the P_L_ change at each insufflation remains constant. Therefore, when the patient breathes spontaneously and generates negative pleural pressure swings, there is a concomitant decrease in the airway pressure waveform, which usually takes an upwardly concave appearance. The difference in the airway pressure between passive and active conditions corresponds to the patient’s Pmus. Therefore, the difference in the areas under the two airway pressure waveforms is equal to the PTPes [61]. Thus, the greater the inspiratory effort, the more the airway pressure decreases during insufflation, which makes it possible to visually detect an intense inspiratory effort (Figure 3).

Patient–ventilator asynchrony can also be assessed. In fact, visual inspection of flow and airway pressure waveforms allows the detection of breath stacking [34,62], regardless of whether it is the consequence of double triggering [34] or reverse triggering [35,36]. The most effective intervention to suppress the occurrence of double triggering is to change the ventilatory mode for pressure support ventilation [33]. By doing this, the tidal volume usually increases above 6 mL/kg of predicted body weight but remains much lower than the volume delivered during a double triggering [33]. If the clinician considers that tidal volume should always be strictly controlled, then the other therapeutic option is to increase sedation and/or paralyze the patient. Analgesics and sedatives should then be used according to their respective effects on breathing pattern and drive [43]: opioids decrease respiratory rate and control elevated respiratory drive when it results from pain, propofol and benzodiazepines decrease respiratory effort but the latter also carry a higher risk of delirium [63,64,65], dexmedetomidine provides sedation, anxiolysis and analgesia without decreasing respiratory drive [66]. Careful adjustment of PEEP during assisted ventilation may also be helpful in achieving protective ventilation. In fact, increasing the PEEP in patients with lung recruitability decreases the amount of atelectatic lung, thus reducing the vertical inspiratory gradient of transpulmonary pressure swings between the nondependent and dependent areas [67]. For a given inspiratory effort, this limits the regional lung stress and the resulting risk of pendelluft, local overstretch and cyclic opening/closing in the nondependent lung. A higher level of PEEP may also decrease the respiratory effort without affecting the respiratory drive [67]. This neuro-muscular uncoupling may be due to the reduce curvature of the diaphragm and changes in its force-length relationship resulting from the increase in end-expiratory lung volume at higher PEEP [68]. Increasing end-expiratory lung volume may also relieve air hunger during low tidal volume ventilation, thus improving patient-ventilator interactions. Lastly, increasing the level of PEEP may attenuate excessive expiratory breaking, which corresponds to a contractile activation of the diaphragm that continues into the expiratory phase and may result in potentially deleterious eccentric load [44]. However, potential protective effects of higher PEEP during assisted ventilation should always be balanced with the risk of overdistension. Future research should assess drug and non-drug strategies to control respiratory drive and effort during assist-control ventilation [43].

#### 6.2.2. Detecting a Risk of P-SILI in Pressure Support Ventilation

During pressure support ventilation, the detection of high tidal volume may easily alert in a patient with severe lung injury. However, in case of low respiratory system compliance and high resistance, detecting a strong inspiratory effort without Pes monitoring may be particularly difficult, as it may not result in high tidal volume despite large P_L_ variations (Figure 2). It may however carry some specific risks, especially in case of important increase in resistance as described previously. Such increase in respiratory resistance may frequently arise in case of secretions clogging the endotracheal tube. Most of the time, the respiratory effort assessment during pressure support ventilation is limited to a clinical evaluation looking for signs of respiratory distress. In case of suspected high inspiratory effort, increasing the pressure support level may properly unload the respiratory muscles [50]. As described above, personalizing the level of PEEP may also prove useful for achieving lung (and diaphragm) protective ventilation during partial ventilatory support. Future research should focus on better assessing the respiratory mechanics and effort during pressure support ventilation.

#### 6.2.3. Detecting a Risk of P-SILI during Noninvasive Strategies

Tonelli et al. recently reported a series of 30 patients under noninvasive ventilation (NIV) for de novo acute hypoxemic respiratory failure in which an esophageal catheter had been inserted [69]. All patients exhibited an excessive inspiratory effort upon NIV initiation. After two hours of NIV, the patients who succeeded NIV had dramatically decreased their respiratory effort as compared to patients who failed NIV. Even though this observation might be related to different ventilator settings adjustments [70], persistently high inspiratory effort could be an early predictor of NIV outcome and may theoretically expose patients to the risk of P-SILI. During NIV for de novo hypoxemic respiratory failure, we additionally reported that: (1) a protective tidal volume (i.e., between 6 and 8 mL/kg of predicted body weight) was impossible to achieve in about 80% of patients despite pressure support level adjustment; and (2) a tidal volume greater than 9.5 mL/kg of predicted body weight was independently associated with NIV failure [12]. These observations were replicated by the post hoc analysis of patients randomized in the NIV arm of the FLORALI study [13]. In this population, a tidal volume greater than 9 mL/kg of predicted body weight was identified as a strong predictor of intubation. Whether the clinical outcome of these patients was partly driven by P-SILI is a matter of debate. In the same population, the FLORALI trial showed a decrease in mortality at Day-90 with the use of high flow nasal cannula oxygen therapy (HFNC) compared to NIV [71]. During HFNC, the continuous washing out of the anatomical dead space [72] yields a better ventilatory efficiency decreasing the need for ventilation at constant PaCO_2_ and pH [73]. Conceptually, HFNC could thus limit the risks of P-SILI compared to NIV. In this regard, the main drawback is the impossible monitoring of the tidal volume. The ROX index, defined as the ratio of SpO_2_/FiO_2_ to respiratory rate, i.e., a ratio of the severity of hypoxemia to a surrogate of respiratory drive, may help prompt recognition of patients at risk of HFNC failure [74]. A ROX index <2.85, <3.47 and <3.85 at two, six and 12 h of HFNC initiation, respectively, are predictors of HFNC failure [74]. NIV delivered through a helmet may potentially offer additional protection against P-SILI. In fact, in a physiological comparison between HFNC and NIV helmet in 15 patients with moderate to severe hypoxemia, Grieco et al. reported that NIV helmet reduced inspiratory effort with similar transpulmonary pressure swings, PaCO_2_, and comfort. In a subsequent multicenter prospective clinical trial comparing HFNC and NIV helmet Grieco et al. randomized 110 COVID-19 patients with moderate to severe hypoxemia and reported a lower endotracheal intubation rate and higher number of days free of invasive mechanical ventilation within 28 days with NIV helmet. During noninvasive ventilatory supports, potential mechanisms of P-SILI are therefore consistently associated with the outcome. Further studies are however still needed to determine the best noninvasive strategy during de novo hypoxemic respiratory failure.

During the COVID-19 pandemic, P-SILI has been much debated, especially when balancing the risk of early versus late intubation [17,18]. Assessment of such risk has generally been biased by the need to use noninvasive strategies in exceptional settings of massive influx of patients and concomitant shortage of ICU beds and mechanical ventilators [75,76,77,78,79]. Anyway, there is no strong evidence from observational studies that timing of intubation have altered the outcome [80,81,82], letting wide open the area of research.

## 7. Conclusions

There is an accumulation of experimental and clinical data tending to support the relevance of P-SILI. However, it is important to note that to date, no clinical study has demonstrated that a ventilatory strategy to limit the risk of P-SILI can improve patients’ outcome. P-SILI is supposed to occur especially when large inspiratory efforts are imposed to severely injured lungs. Transposing this concept into clinical practice requires the assessment of the patient’s inspiratory effort and the detection of potentially hazardous patient-ventilator interactions, which may be greatly facilitated by Pes monitoring. If Pes is unavailable, careful clinical assessment and analysis of tidal volume, flow and airway pressure waveforms from the ventilator can help detecting situations at risk of P-SILI. Future research should particularly focus on P-SILI monitoring and better defining the preventive strategy during both noninvasive and invasive ventilatory supports. Until then, a clinical reasoning taking into account the amount of respiratory drive and effort, the severity of the underlying lung damage, the patient’s evolutionary profile, and the systematic search for causes of increased respiratory drive independent of the respiratory load, such as metabolic acidosis or uncontrolled pain, must help personalizing treatment. In some situations, controlled mechanical ventilation may become a protective treatment.

## Data Availability

No new data were created or analyzed in this study. Data sharing is not applicable to this article.
